# Bioactive Constituents of *Zanthoxylum rhetsa* Bark and Its Cytotoxic Potential against B16-F10 Melanoma Cancer and Normal Human Dermal Fibroblast (HDF) Cell Lines

**DOI:** 10.3390/molecules21060652

**Published:** 2016-05-24

**Authors:** Ramesh Kumar Santhanam, Syahida Ahmad, Faridah Abas, Intan Safinar Ismail, Yaya Rukayadi, Muhammad Tayyab Akhtar, Khozirah Shaari

**Affiliations:** 1 Laboratory of Natural Products, Institute of Bioscience, Universiti Putra Malaysia, UPM Serdang 43400, Selangor, Malaysia; rameshcra@gmail.com (R.K.S.); faridah_abas@upm.edu.my (F.A.); safinar@upm.edu.my (I.S.I.); yaya_rukayadi@upm.edu.my (Y.R.); tayyabakhtar@hotmail.com (M.T.A.); 2 Faculty of Biotechnology and Biomolecular Sciences, Universiti Putra Malaysia, UPM Serdang 43400, Selangor, Malaysia; syahida@upm.edu.my

**Keywords:** *Zanthoxylum rhetsa*, lignans, triterpenes, alkaloids, cytotoxicity, NMR spectroscopy, GC-MS

## Abstract

*Zanthoxylum rhetsa* is an aromatic tree, known vernacularly as “Indian Prickly Ash”. It has been predominantly used by Indian tribes for the treatment of many infirmities like diabetes, inflammation, rheumatism, toothache and diarrhea. In this study, we identified major volatile constituents present in different solvent fractions of *Z. rhetsa* bark using GC-MS analysis and isolated two tetrahydrofuran lignans (yangambin and kobusin), a berberine alkaloid (columbamine) and a triterpenoid (lupeol) from the bioactive chloroform fraction. The solvent fractions and purified compounds were tested for their cytotoxic potential against human dermal fibroblasts (HDF) and mouse melanoma (B16-F10) cells, using the MTT assay. All the solvent fractions and purified compounds were found to be non-cytotoxic to HDF cells. However, the chloroform fraction and kobusin exhibited cytotoxic effect against B16-F10 melanoma cells. The presence of bioactive lignans and alkaloids were suggested to be responsible for the cytotoxic property of *Z. rhetsa* bark against B16-F10 cells.

## 1. Introduction 

Skin is the largest organ of the body, protecting it from many external stresses such as radiation, temperature, chemicals and microbes. It comprises the epidermis (outer layer), dermis (inner layer) and subcutaneous tissues. The dermis includes sweat glands, hair follicle, blood vessels and nerves, while the epidermis is made up of three cells namely squamous, basal and melanocytes [1]. Under normal conditions, these cells undergo systematic cell division and produce daughter cells. However, in case of abnormal cell division, the epidermal cells grow aberrantly and can cause three types of skin carcinomas based on their originating cells *i.e.*, squamous, basal and melanoma skin cancers. The squamous and basal skin cancers, which are non-melanoma cancers, are common and not life threatening. These types of non-melanoma skin cancers can be cured if treated at an early stage. Clinical data show that skin cancer occurs primarily based on geographical areas. Basal cell carcinoma (BCC) is usually reported among Hispanics, Caucasians, Japanese and Chinese Asian, whereas squamous cell carcinoma (SCC) is more common among African, Americans and Asian Indians [2]. In comparison, melanoma cancer is less common but life threatening. Malignant melanoma is the nineteenth most commonly reported cancer in the world, and the seventh most common in the United States (US) [3]. The rate of incidence of melanoma cancer has risen exponentially as shown by the 2015 statistics that reported 73,870 cases (42,670 men and 31,200 women) as being diagnosed with the disease in the US alone [4]. Meanwhile, a total of 12,960 (7640 men and 5320 women) new cases were reported in Australia, for the year 2015. The number is expected to increase to 17,570 by 2020 [5]. The factors influencing this type of carcinoma are fair skin, a history of sunburn, continuous exposure to UV or other radiation, moles, heredity or weak immune system, *etc*. Current methods of treatment for melanoma cancers include cryotherapy, external surgery, radiation therapy, chemotherapy, photodynamic therapy, biological therapy and targeted drug therapy such as with vemurafenib, dabrafenib, and trametinib [2]. All of these methods of treatment have certain limitations such as high costs, side effects and reoccurrence. Thus, safer alternative remedies for skin cancer need to be found. In this context, traditional medicine and medicinal plants, in general, offer an excellent resource for the identification of new therapeutic agents for use against diseases, including skin cancer.

*Zanthoxylum rhetsa* (Roxb.) DC (syn. *Zanthoxylum budrunga*, Fam. Rutaceae) is a medium-sized aromatic tree with conical prickles on the bark of the trunk and branches. It is widely distributed in the tropical and sub-tropical regions, including India, Bangladesh, Indonesia, China and Malaysia [6]. The plant has long been valued for its medicinal uses. The Kannikar tribes from Tamil Nadu utilized a paste made from the prickly thorns of *Z. rhetsa* to treat breast pain and to increase lactation in breastfeeding mothers. The plant shoots are consumed as a vegetable by the Adi tribes of Arunachal Pradesh, India [7]. Meanwhile, various parts of *Z. rhetsa* are traditionally used as an aromatic, astringent, antimicrobial, antiseptic and antidiabetic agent, as well as used to treat snake bites, inflammatory dermatosis, cholera, rheumatism, and toothache [8,9]. Characteristic secondary metabolites of *Zanthoxylum* species include lignoids, alkaloids, amides, flavonoids, terpenes, sterols and coumarins [10]. Alkaloids are abundant in the trunk and root bark, and are typically of the isoquinoline and quinolone types. Lignoids are also abundant in the genus, typically of the diarylbutirolactones and furofuranic types. Previous phytochemical investigations on *Z. rhetsa* have shown the presence of a variety of compounds including monolignols, coumarins, alkaloids and lignans namely 3,5-dimethoxy-4-geranyloxycinnamyl alcohol, xanthyletin, 8-methoxy-*N*-methylflindersine and sesamin [11] as well as zanthorhetsamide [12]. Ahsan *et al.* [13] further reported quinolone terpene alkaloids namely, chelerybulgarine, 2′-episimulanoquinoline, 2,11-didemethoxyvepridimerine B, and rhetsidimerine, from the root bark of the plant. Moreover, GC-MS analysis of the ethanol extract of *Z. rhetsa* spines revealed fifteen compounds, of which 1,2-benzenedicarboxylic acid and diisooctyl ester were the major components, followed by oleic acid and n-hexadecanoic acid [14]. Likewise, the volatile constituents of the fruits, seed coat and leaf were also identified through GC-MS, wherein, sabinene, carophyllene oxide, spathulenol, α-pinene, 4-terpineol, 3-elemene, β-phellandrene, 3-pinene, γ-terpinene and myrcene were the predominant compounds [15,16,17]. The stem bark of *Z. rhetsa* has been shown to possess anti-inflammatory activity, which was mediated by down-regulation of TNF-α, mRNA expression of pro-inflammatory cytokines and also by inhibition of iNOS and COX-2 production [18]. The seeds of the plant have been reported to have sunscreening properties [19]. Previously, we disclosed the Ultraviolet A/Ultraviolet B (UVA/UVB) protecting properties of the bark extracts of *Z. rhetsa* [20]. Although previous studies have revealed the anti-inflammatory and other therapeutic activities of the plant extract, to the best of our knowledge, the potential of *Z. rhetsa* bark extract to protect against skin cancer has not been investigated. In this study we publicize the results of our investigation on the cytotoxic properties of *Z. rhetsa* bark extract, against Human Dermal Fibroblasts (HDF) and B16-F10 melanoma cells. We also report the isolation and identification of four bioactive constituents from the chloroform fraction of the *Z. rhetsa* bark extract.

## 2. Results and Discussion

### 2.1. Identification of Bioactive Compounds Through GC-MS Analysis

The GC-MS spectra for the crude methanol extract and the solvent fractions of *Z. rhetsa* bark are shown in Figure S1. The compounds present in the active fractions were identified by matching their recorded mass spectra with those retrieved from the NIST11 and WILEY229 mass spectral libraries, and by comparison with literature values [21,22,23,24,25]. Overall, a total of thirty-nine compounds were identified from the bark of *Z. rhetsa*. The identified compounds and their mass data are tabulated in Table 1.

Identification of the tetrahydrofuran lignans was based on their characteristic MS fragmentations via *m*/*z* 219 [(CH_3_O)_2_-C_6_H_3_-(CH_2_)_2_-(CH)_2_-O-CH_2_]^+^, 177 [(CH_3_O)_2_-C_6_H_3_-CH-CH=CH2]^+^, 165 [(CH_3_O)_2_-C_6_H_3_-CO]^+^ [26,27]. Previous studies also revealed the presence of alkaloids and lignans in this species [11,13]. To the best of our knowledge, this is the first report on the presence of compounds such as kobusin, yangambin, *epi*-eudesmin, eudesmin, 8-hydroxy-4′-methoxypinoresinol, hesperetin, magnolin reticuline, allocryptopine, usambanoline, dihydronitidine, *N*-methyllaurotetanine and chelerythrine in the bark of *Z. rhetsa.*

### 2.2. Evaluation of Cytotoxic Activity 

MTT assay was performed to evaluate the toxic effects of the crude methanolic extract, solvent fractions and the isolated compounds, against HDF and B16-F10 melanoma cells. The extract and solvent fractions showed minimal effect on HDF cell and exhibited no toxicity against normal skin cells (Figure S2).

**Figure 1:**
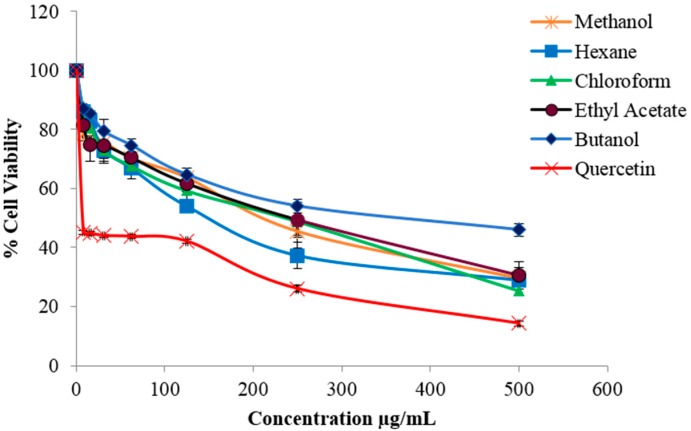
Cytotoxic effect of various fractions of *Z. rhetsa*, at different concentrations (0–500 µg/mL), against B16-F10 melanoma cells. Data are expressed as mean ± SD of three independent experiments. Quercetin was used as the positive control.

Other *Zanthoxylum* species have also been reported to be non-toxic to normal cell lines [28,29]. Meanwhile, the crude methanolic extract and solvent fractions were found to be toxic towards B16-F10 cells, causing significant cell death (Figure 1).The chloroform fraction exhibited the strongest cytotoxic effect followed by hexane, ethyl acetate, methanol and butanol fractions with IC_50_ values 156, 132.7, 174.3, 168 and 263.1 µg/mL, respectively.

GC-MS analysis showed that the crude methanolic extract and all the solvent fractions of *Z. rhetsa* bark contained a diverse array of constituents, consisting of monolignols, coumarins, alkaloids and tetrahydrofuran lignans. The GC-MS result for the hexane fraction (Table 1) showed lupeol (36.9%) to be the major constituent along with lower amounts of other constituents comprising simple ketones, sesquiterpenes, fatty acids, sterols, triterpenes and tetrahydrofuran lignans. The GC-MS result for the chloroform fraction (Table 1) showed the tetrahydrofuran lignans, yangambin (30.4%) and eudesmin (26.7%), as major constituents. Other tetrahydrofuran lignans were also observed as minor constituents together with simple ketones, triterpenes and isoquinoline alkaloids. The ethyl acetate fraction contained coniferyl alcohol (21.5%) as the major constituent along with other monolignols, coumarins, isoquinoline alkaloids and lignans. In the butanol fraction, quinolone alkaloids were predominant along with ketones, monolignols and several unknown compounds. The difference in the chemical constituents between the solvent fractions could be responsible for the difference in their cytotoxic properties as revealed by the MTT assay. Ahsan and co-workers reported that dimeric quinolone-terpene alkaloids isolated from *Z. rhetsa* root bark showed weak cytotoxic effect against six stomach cancer cell lines [13]. Meanwhile, according to Mukhija and his co-workers , tetrahydrofuran lignans isolated from the petroleum ether extract of *Z. alatum* bark were cytotoxic against lung and pancreatic carcinoma cell lines [24]. These studies suggested that lignan-rich fractions exert more cytotoxic effect in comparison to fractions rich in quinolone-terpene alkaloids. In another study, a structure-activity relationship analysis on the bioactive tetrahydrofuran lignans of *Z. planispinum* root, revealed that the phenolic groups in the lignans were responsible for the increase in cytotoxicity against human tumour cell lines [30]. Thus, based on these previous findings, it is highly probable that the cytotoxic property of the chloroform and hexane fractions of *Z. rhetsa* were due to the presence of the tetrahydrofuran lignans.

### 2.3. Structural Identification of the Isolated Compounds

**Figure 2:**
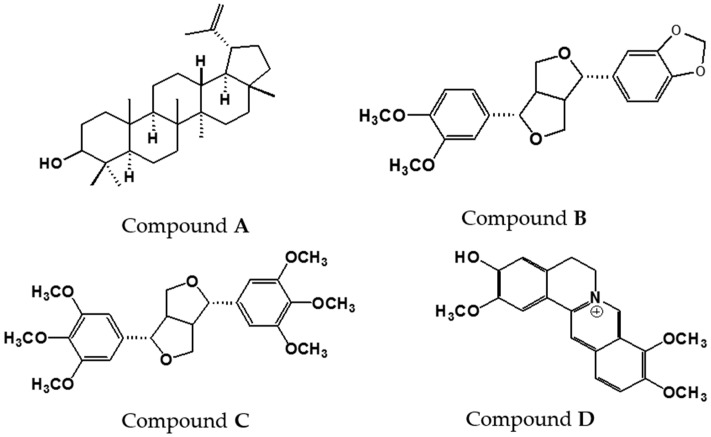
Structures of compounds isolated from the chloroform fraction of *Z. rhetsa* bark.

Compounds **A**–**D**, isolated from the bioactive chloroform fraction are shown in Figure 2. The structures of the compounds were elucidated using 1D and 2D NMR and mass spectral data as well as comparison with literature values. The physical and spectroscopic data of the compounds are listed as follows:

Compound **A** (White powder): Melting point: 215–216 °C, EI-MS: *m*/*z* 426 [M]^+^ (calc. for C_30_H_50_O, 426.71). ^1^H-NMR (500 MHz, CDCl_3_) δ: 0.76, 0.79, 0.83, 0.95, 0.97, 1.03, 1.68 (3H, s, 7 *×* CH_3_), 3.18 (1H, dd, *J* = 5.5, 10.7 Hz, H-3), 4.57 (1H, s, H-29a), 4.69 (1H, s, H-29b). ^13^C-NMR (125 MHz, CDCl_3_) δ: 151.00 (C-20), 109.3 (C-29), 79.0 (C-3), 55.3 (C-5), 50.4 (C-9), 48.3 (C-18), 48.0 (C-19), 43.0 (C-17), 42.8 (C-14), 40.8 (C-8), 40.0 (C-22), 38.9 (C-13), 38.7 (C-4), 38.0 (C-1), 37.2 (C-10), 35.6 (C-16), 34.3 (C-7), 29.8 (C-21), 28.0 (C-23), 27.4 (C-12), 25.1 (C-2), 20.9 (C-11), 19.3 (C-30), 18.3 (C-6), 18.0 (C-28), 16.1 (C-25), 16.0 (C-24), 15.4 (C-27), 14.5 (C-26). Compound **A** was identified as lupeol [31].

Compound **B** (Colourless gum): EI-MS: *m*/*z* 370 [M]^+^ (calc. for C_21_H_22_O_6_, 370.39). ^1^H-NMR (500 MHz, CDCl_3_) δ: 6.91–6.77 (6H, m, aromatic rings), 5.95 (2H, s, OCH_2_O-benzo-1,3-dioxole moieties), 4.73 (2H, t, *J* = 10.5 Hz, H-2/6, tetrahydrofuran moieties), 4.26 (2H, d, *J* = 9.0 Hz, H-4b/8b, equatorial protons-tetrahydrofuran moieties, 3.89 (m, 2H, H-4a/8a, axial protons-tetrahydrofuran moieties), 3.80 (s, 3H, OCH_3_), 3.87 (s, 3H, OCH_3_), 3.08 (m, 2H, H-1/5 tetrahydrofuran moieties) ^13^C-NMR (125 MHz, CDCl_3_) δ: 149.2 (C-3′), 148.6 (C-4′), 147.8 (C-3′′), 147.1 (C-4′′), 135 (C-1′′), 133.2 (C-1′), 119.3 (C-6′′), 118.2 (C-6′), 111.1 (C-5′), 109.2 (C-2′), 108 (C-5′′), 106.4 (C-2′′), 100.9 (-OCH_2_O-), 85.8 (C-2), 85.7 (C-6), 71.7 (C-4), 71.6 (C-8), 56 (-OCH_3_), 55.9 (-OCH_3_), 54.3 (C-5), 54.2 (C-1). Compound **B** was identified as kobusin [32].

Compound **C** (White needles): Melting point: 119–121 °C, EI-MS: *m*/*z* 446 [M]^+^ (calc. for C_24_H_30_O_8_, 446.49). ^1^H-NMR (500 MHz, CDCl_3_) δ: 6.57 (4H, s, H-2′/6′, 2′′/6′′, aromatic rings), 4.75 (2H, d, *J* = 3.7 Hz, H -2/6, tetrahydrofuran moieties), 4.31 (2H, dd, *J*_1_ = 6.4 Hz, *J*_2_ = 8.6 Hz, H-4b/8b, equatorial protons-tetrahydrofuran moieties), 3.93 (2H, dd, *J*_1_ = 2.7 Hz, *J*_2_ = 9.3 Hz, H-4a/8a, axial protons-tetrahydrofuran moieties), 3.88 (12H, s, OCH_3_), 3.84 (6H, s, OCH_3_), 3.11 (2H, m, H-1/5) ^13^C-NMR (125 MHz, CDCl_3_) δ: 153.4 (C-3/3′, 5/5′), 137.5 (C-4/4′), 136.7 (C-1/1′), 102.9 (C-2/2′, 6/6′), 85.9 (C-7/7′), 72.1 (C-9/9′), 60.8 (C-4/4′, OCH_3_), 56.2 (C-3/3′, 5/5′, OCH_3_), 54.4 (C-8/8′). Compound **C** was identified as yangambin [33].

Compound **D** (Yellow powder): Melting point: 280–282 °C, EI-MS: *m*/*z* 338.4 [M]^+^ (calc. for C_20_H_20_NO_4_^+^, 338.37). ^1^H-NMR (500 MHz, CDCl_3_) δ: 9.72 (1H, s, H-8), 8.75 (1H, s, H-13), 8.09 (1H, d, *J* = 8.8 Hz, H-12), 7.98 (1H, d, *J* = 8.8 Hz, H-11), 7.65 (1H, s, H-1), 6.86 (1H, s, H-4), 4.91(2H, t, *J* = 6.3 Hz, H-6), 4.20 (3H, s, C-10, OCH_3_), 4.10 (3H, s, C-9, OCH_3_), 4.02 (3H, s, C-3, OCH_3_), 3.20 (2H, t, *J* = 6.4 Hz, H-5) ^13^C-NMR (125 MHz, CDCl_3_) δ: 150.2 (C-3), 148.3 (C-2), 144.6 (C-8), 144.3 (C-10), 138.9 (C-14), 134 (C-12a), 128.9 (C-4a), 126.7 (C-11), 122.9 (C-12), 121.7 (C-8a), 119.4 (C-13), 117.8 (C-14a), 114.5 (C-1), 108.6 (C-4), 61.1 (9-OCH_3_), 56.3 (10-CH_3_), 56.0 (C-6), 55.5 (3-OCH_3_), 26.3 (C-5). Compound **D** was identified as columbamine [34].

*Zanthoxylum* species, especially the bark material, have been previously reported to be rich in tetrahydrofuran lignans. These included six lignans from *Z. nitidium* bark [21], twelve lignans from *Z. schinifolium* stem [22], seven lignans from *Z. armatum* bark [25], three lignans from *Z. budrunga* bark [35], and eight lignans from the roots of *Z. planispinum* [30]. Apart from lignans, various alkaloids have also been reported to be abundant in the bark of several *Zanthoxylum* species. For example, the alkaloids *N*-methyl corydine, magnoflorine and berberine have been reported to be major constituents in the stem and branches of *Z. punctatum* and *Z. monophyllum* [36]. Previous studies on *Z. rhetsa* have also reported the presence of different types of alkaloids. This included 6-acetonyldihydrochelerythrin and arnottianamide in the conical prickles, and the quinolone-terpene alkaloids chelerybulgarine, 2,11-didemethoxyvepridimerine B, rhetsidimerine, simulanoquinoline and 2′-episimulanoquinoline in the root bark [13,37]. In addition to these compounds, the current study further reports the occurrence of kobusin, yangambin and columbamine in the bark of *Z. rhetsa*.

### 2.4. Cytotoxicity Evaluation of Isolated Compounds

**Figure 3:**
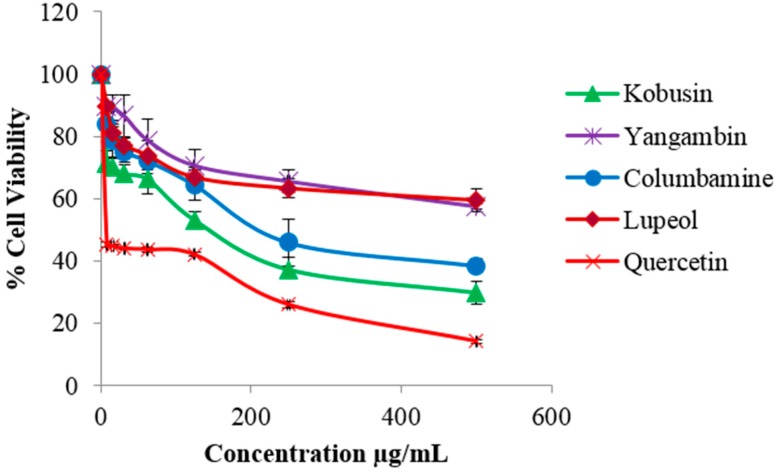
Cytotoxic effect of isolated compounds from *Z. rhetsa* at various concentrations (0–500 µg/mL) against B16-F10 melanoma cells. Data are expressed as mean ± SD of three independent experiments. Quercetin was used as the positive control.

The isolated compounds were evaluated for cytotoxicity against HDF and B16-F10 melanoma cells. The isolated compounds were found to be non-toxic to HDF cells (Figure S3). However kobusin caused a high percentage of cell death against B16-F10 cells followed by columbamine, lupeol and yangambin, giving IC_50_ values of 112.2, 195.6, 377.8 and 442.4 µg/mL, respectively. Quercetin, the positive control, gave an IC_50_ value of 22.72 µg/mL. The results of the cytotoxicity test on the isolated compounds are shown in Figure 3. These results were in good agreement with previous reports where kobusin, isolated from *Z. alatum* bark, showed strong cytotoxic effect, with IC_50_ values of 34.71 µg/mL and 32.86 µg/mL, against A549 (lung) and MIA-PaCa (pancreatic) cell lines, respectively [24]. On the other hand, columbamine, previously isolated from *Rhizoma coptidis*, has been found to be moderately cytotoxic with an IC_50_ value of 226.54 µg/mL against HepG2 cells [38]. Meanwhile, lupeol, isolated from *Grewia tiliaefolia*, was reported to be weakly cytotoxic with an IC_50_ value of 330 µg/mL against B16-F10 cells [39]. It was also found to mediate anticancer activity against malignant melanoma cells by altering the level of Bcl-2, Bax protein and Wnt/β-catenin signaling [40]. In the case of yangambin, previous report of its cytotoxic effect had only been reported on murine macrophages. The toxic effect was found to be low. Structure-activity relationship studies have associated the higher cytotoxic effect of kobusin and columbamine with the presence of phenylmethylenedioxy and phenolic groups, respectively [30,38].

## 3. Materials and Methods 

### 3.1. General Experimental Procedures

Melting points were recorded on a Koffler hot-stage apparatus (Electrothermal 9100, Dubuque, IA, USA) and were uncorrected. GC-MS analysis were carried out on a QP-2010 Ultra GCMS spectrometer (Shimadzu, Kyoto, Japan) equipped with a flame ionization detector (FID). 1D and 2D NMR spectra were recorded on a Unity INOVA 500 MHz spectrometer (Varian, Palo Alto, CA, USA) using standard pulse programs. Chloroform-*d* (CDCl_3_) and methanol-*d*_4_ (CD_3_OD) were used as NMR solvents, and TMS was utilized for internal referencing. Solvents used for extraction and isolation were of analytical grade and obtained from R & M chemicals (Edmonton, AB, Canada). Kieselgel 60 (0.040–0.063 mm) and Lichroprep RP-18 (40–63 µm) were purchased from Merck (Darmstadt, Germany) while Sephadex LH-20 was purchased from Sigma (St. Louis, MO, USA). Dulbecco’s Modified Eagle Medium (DMEM), fetal bovine serum (FBS), trypsin–EDTA, penicillin/streptomycin were purchased from GIBCO (Lifetechnologies, Grand Island, NY, USA). Trypan blue and MTT (3-(4,5-dimethylthiazole-2-yl)-2,5-biphenyl tetrazolium bromide) were purchased from Sigma. A SpectraMax Plus (Molecular Devices, Sunnyvale, CA, USA) microplate reader was used in the bioassays.

### 3.2. Sample Collection

The trunk barks of *Z. rhetsa* were collected from Pangkor Island, Malaysia. A voucher specimen (SK2226/13) was deposited at the Herbarium of the Institute of Bioscience, Universiti Putra Malaysia.

### 3.3. Extraction

The air dried, powdered bark material (910 g) of *Z. rhetsa* was subjected to ultrasound-assisted extraction with 100% methanol and dried under vacuum at 40 °C to yield the crude methanolic extract (65 g). The extract was then subjected to solvent-solvent extraction using organic solvents of increasing polarities. The resulting solvent fractions were dried under vacuum, lyophilized to yield 14 g hexane, 17.4 g chloroform, 2 g ethyl acetate and 5.3 g butanol fractions. All the samples were stored at −20 °C prior to further analysis [20].

### 3.4. Cell Culture

Both HDF and B16-F10 cells were provided by the Laboratory of Vaccines and Immunotherapeutics (LIVES), Institute of Bioscience, UPM. Human dermal fibroblasts (HDF) cells (passage 11–15) were maintained in DMEM medium containing 5% fetal bovine serum, 1% penicillin (100 U/mL) and streptomycin (100 μg/mL). Mouse melanoma (B16-F10B) cells (passage 11–15) were also maintained in DMEM medium with 10% fetal bovine serum, 1% penicillin (100 U/mL) and streptomycin (100 μg/mL). Cells were sub-cultured, incubated and maintained in a humidified 5% CO_2_ incubator at 37 °C. Only the cells that attained more than 80% confluence (growth phase) were used for cell seeding [41].

### 3.5. MTT Proliferation Assay 

Cells were seeded in 96 well plates (1 × 10^4^ cells/well) and incubated in 5% CO_2_ incubator at 37 °C for 24 h. The cells were treated with serial dilutions of *Z. rhetsa* extract and positive control ranging from 500, 250, 125, 62.5, 31.25, 15.625, 7.81 µg/mL. The cells treated with medium without the sample served as negative control. Quercetin which is known to inhibit cell growth, induce apoptosis and is cytotoxic against B-16 melanoma cells, served as positive control [42,43]. All the samples and positive control were dissolved in DMEM medium with a small amount of DMSO (the final concentration of DMSO was lower than 0.1%). Negative control was also treated with the same solvent vehicle without the sample. After 24 h, cell viability was analyzed using MTT assay wherein 20 µL of MTT (5 mg/mL PBS) was added to each well and incubated at 37 °C for 3–4 h. MTT enters the mitochondria of the viable cells and gets reduced to insoluble formazan (dark purple) product. The formazan product was pelleted by centrifuging the plates at 1200 rpm for 5 min. Afterwards, the cells were treated with DMSO to dissolve the formazan product and subsequently its absorbance was measured spectrophotometrically after 30 min at 570 nm, using microplate reader (SpectraMax Plus). All experiments were performed in triplicates with three different passage cells:

Cell Viability (%) = (Treated cells/Untreated cells) × 100
(1)

### 3.6. GC-MS Analysis of Solvent Fractions

The crude methanolic extract and the solvent fractions were subjected to GC-MS analysis using a BPx5 column (30 m × 0.25 mm × 0.25 µm). The oven temperature program was set to 50 °C for 0 min, heated to 330 °C at the rate of 3 °C/min and held constant at 330 °C for a further 5 min. Helium was used as carrier gas with the following conditions: total flow—11.8 mL/min, column flow—0.8 mL/min, linear velocity—32.4 cm/s, purge flow—3.0 mL/min, split ratio—10. Mass spectra were recorded with ion source temperature of 200 °C and interface temperature of 250 °C. The mass scan parameters included a start time of 2.5 min and end time of 95.0 min. The acquisition (ACQ) parameters were as follows: Scan event time—0.10 s, scan speed—10000, mass range—40 *m*/*z* to 700 *m*/*z*.

### 3.7. Isolation of Chemical Constituents from the Bioactive Chloroform Fraction 

**Figure 4:**
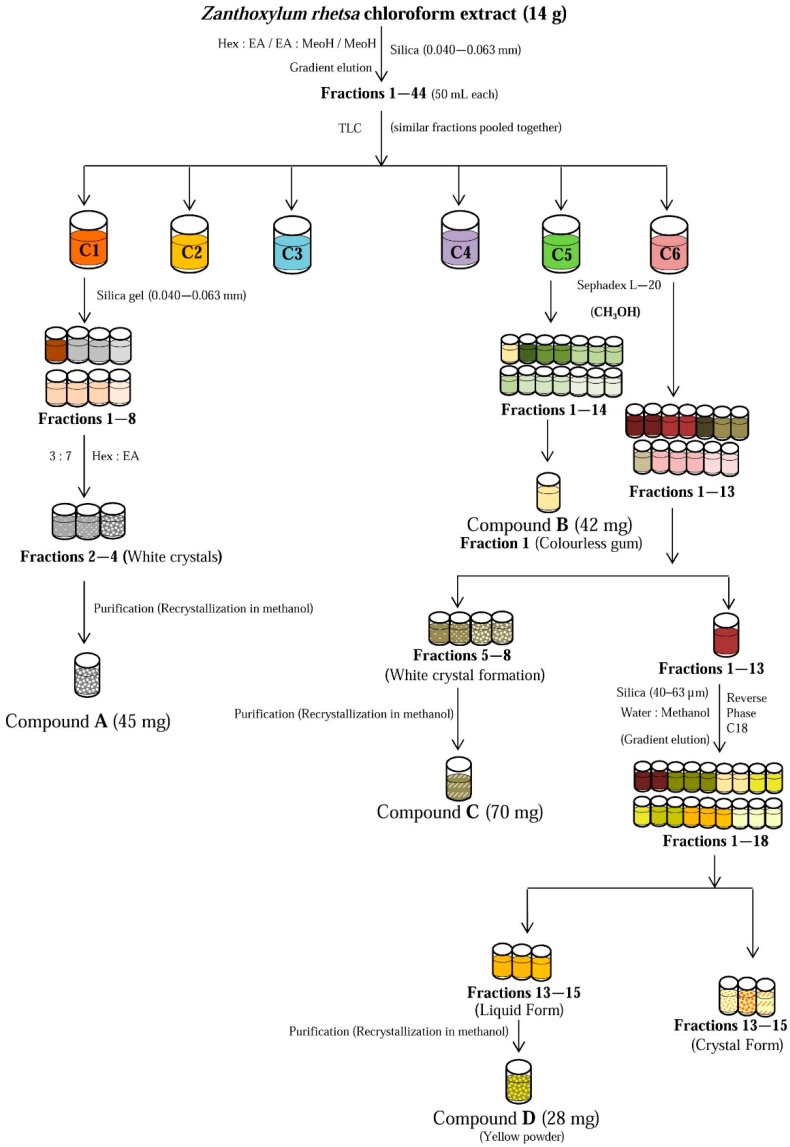
Isolation of compounds from the chloroform fraction of *Z. rhetsa* extract using column chromatography.

The scheme for the isolation of chemical constituents from the chloroform fraction is illustrated in Figure 4.

The dried chloroform extract (14 g) was subjected to column chromatography (CC) over silica gel (0.040–0.063 mm) with solvent systems hexane:ethyl acetate (9:1, 8:2 7:3, 6:4, 5:5, 0:10) followed by ethyl acetate:methanol (9:1) and 100% methanol, to give 44 sub-fractions (~50 mL each). Sub-fractions with similar TLC profiles were pooled together and relabeled as C1, C2, C3, C4, C5, C6. Compound **A** (lupeol) was isolated as white powder (45 mg) from sub-fraction C1 using normal phase CC, where hexane and ethyl acetate were used as eluent at the ratio of 3:7. Compound **B** (42 mg, kobusin) was isolated from sub-fraction C5 by first passing it through a Sephadex LH-20 column, eluted with methanol. Sub-faction C6 was similarly pretreated with Sephadex LH-20 to give thirteen sub-fractions. White needle like crystals were filtered from sub-fractions 5–8, which yielded 70 mg of Compound **C** (yangambin) after further purification by recrystallization in methanol. The remaining sub-fraction C6 was subjected to CC over reverse phase silica (40–63 µm), eluted with solvent system water:methanol at varying ratios (9:1, 7:3, 5:5, 4:6, 3:7, 0:10), to give 18 sub-fractions. Sub-fractions 13–15 were pooled and further purified via repeated CC and recrystallization steps to yield 28 mg of Compound **D** (columbamine).

### 3.8. Statistical Analysis

Data were expressed as mean ± SD of three independent experiments. Statistical analysis were performed through GraphPad Prism software Version 5 using one way ANOVA followed by Turkey’s t- test. Differences were considered to be significant when *p* values < 0.05.

## 4. Conclusions

Our findings suggest that the presence of compounds such as lignans and alkaloids plays an important role in the overall cytotoxic effects of Z. *rhetsa* bark extract towards the melanoma B16-F10 cell line. In contrast, all the solvent fractions of the bark extract and the isolated compounds were found to be relatively non-toxic to HDF cells. The selective cytotoxicity of the extract and isolated compounds may be due to the metabolic defects in the cancerous cells or the peculiar reaction of the compounds against the melanoma cells [44,45,46,47]. Nevertheless, the mechanism of action underlying the selective cytotoxicity is worthy of further study to fully understand the exact mechanism. Overall, the results of this study indicated that *Z. rhetsa* bark extract may have potential use as a dermo protective ingredient in skincare or cosmeceutical products.

## Figures and Tables

**Table 1:** Compounds identified in various solvent fractions of *Z. rhetsa* using GC-MS analysis.

Sample	Compound Name	Retention Time	MW	*m*/*z* Fragments	Area (%)
**Methanol crude extract**	2-Undecanone	24.33	170	58, 38, 43, 71	1.984
	Coniferyl alcohol	43.672	180	137, 124, 91	0.936
	Scoparone	52.2	206	191, 178, 163, 131	4.104
	Reticuline (+)	75.2	329	192,177	5.111
	Allocryptopine	80.9	369	283, 206, 164	2.038
	Unknown	81.38	343	58	13.87
	*epi*-Eudesmin	82.787	386	177, 165, 151, 107	2.018
	Sesamin	84.25	354	323, 203, 161, 149	3.731
	Kobusin	85.052	370	339, 219, 203, 165, 149	3.488
	Eudesmin	85.823	386	355, 219, 165,151	17.57
	8-Hydroxy-4′-methoxy-pinoresinol	86.502	388	357, 339, 208, 165,151	0.642
	β-Amyrin	87.8	426	218, 203, 189	1.197
	Mangnolin	88.25	416	219, 207, 195, 165, 151	5.337
	Lupeol	88.91	426	411, 315, 218, 207,189	13.74
	Yangambin	90.65	446	415, 224,195,181	24.24
**Hexane**	2-Undecanone	24.35	170	71, 38, 43	13.11
	2-Tridecanone	33.373	198	71, 58, 43	2.662
	Farnesol	42.26	222	136, 93, 81, 69	9.583
	Hexadecanoic acid	49.7	270	227, 143, 87, 74, 57	2.1
	9-Octadecenoic acid-(*Z*)-methyl ester	55.47	296	264, 222, 180, 97, 55	1.349
	Germacrene	83.66	204	189, 161, 121, 107, 69	1.33
	Sesamin	84.25	354	323, 219, 203, 149	3.622
	Kobusin	85.065	370	339, 219, 203, 165, 149	2.342
	Eudesmin	85.825	386	355, 219, 165, 151	8.488
	Stigmast-5-en-3-ol	86.75	414	396, 381, 329, 213, 107	0.735
	9,12-Octadecadienoic acid	87.4	279	204, 189, 161, 136, 121, 69	4.182
	β-Amyrin	87.82	426	218, 203, 189	5.597
	Mangnolin	88.26	416	219, 207, 195, 177, 165	2.304
	Lupeol	89.03	426	411, 315, 218, 207, 189	36.94
	Yangambin	90.65	446	415, 224, 195, 181	5.659
**Chloroform**	Scoparone	52.27	206	191, 178, 163 , 135	7.343
	Sinapyl alcohol	52.69	210	182, 167, 149,107	1.468
	Allocryptopine	80.922	369	283, 206, 164, 149	1.717
	Usambanoline	82.85	386	204, 189, 151	3.822
	Sesamin	84.31	354	323, 203, 161, 149	5.913
	Unknown	84.51	506	181,182, 151	2.13
	Dihydronitidine	84.67	349	333, 304, 290, 204, 149	0.401
	Kobusin	85.14	370	339, 219, 203, 165, 149	6.04
	*Epi*-eudesmin	85.52	386	372, 219, 194, 165, 151	0.356
	Eudesmin	85.96	386	355, 219, 194, 177, 165, 151	26.75
	8-Hydroxy-4′-methoxy-pinoresinol	86.56	388	357, 339, 208, 194, 165, 151	1.398
	Mangnolin	88.37	416	385, 219 , 207, 195, 177, 165, 151	8.118
	Lupeol	88.91	426	411, 315, 218, 207,189	3.55
	Yangambin	90.82	446	415, 224,195,181	30.38
**Ethyl acetate**	2,2-Dimethoxybutane	3.475	118	103. 87, 55	1.091
	4-vinylsyringol	36.521	180	165, 137 122	6.444
	Unknown	38.86	-	143, 95,59	1.258
	Coniferyl alcohol	43.645	180	137, 124, 91	24.172
	Butyl 4-hydroxybenzoate	48.66	194	138, 121	2.818
	Benzeneacetic acid	51.42	166	107, 69	1.745
	Unknown	51.88	223	179, 123, 81	0.797
	Sinapyl alcohol	52.6	210	182, 167, 149, 121	7.073
	4-[(6,7-Dimethoxy-1,2,3,4-tetrahydro-1-isoquinolinyl)methyl]phenol	53.52	299	213	1.88
	Quinic acid	59.44	191	123,107, 69	1.249
	Hesperetin	80.54	302	179, 150, 137	1.374
	Allocryptopine	80.79	369	283, 206, 164, 149	5.706
	Sesamin	84.1	354	203,149,161, 121, 103	1.63
	Dihydronitidine	84.52	349	332, 290, 102, 204, 167	6.43
	Kobusin	84.89	370	219, 203,177, 165, 149	3.041
	Eudesmin	85.59	386	189,165, 151,	10.778
	Mangnolin	88.07	416	219, 207, 195, 165, 151	5.125
	Lupeol	88.65	426	412, 315, 218, 207,189	3.187
	Unknown	90.01	388	226, 207, 193, 181	1.819
	Yangambin	90.38	446	415, 224,195,181	12.383
**Butanol**	1-Butanol	2.62	74	56, 43	41.59
	2-Butoxyethanol	7.02	118	87, 57	1.394
	4-vinylsyringol	36.588	180	165, 137, 122	1.007
	Coniferyl alcohol	43.69	180	137, 124, 109	0.815
	Sinapyl alcohol	52.673	210	182, 167, 121, 107	0.565
	(−)-1,2,3,4-Tetrahydro-isoquinolin-6-ol-1-carboxylic acid	72.31	192	177, 148,	2.142
	Unknown	74.678	343	58	3.548
	(+)-Reticuline	75.27	329	192, 177	14.04
	Unknown	76.69	327	58	1.993
	Unknown	78.26	313	58	3.885
	Allocryptopine	80.94	369	283, 206, 164, 149	3.029
	*N*-Methyllaurotetanine	81.165	341	326, 310, 206, 164	0.152
	Unknown	91.6	343	-	25.01
	Nitidine	84.653	349	332,304, 290	0.208
	Chelerythrine	85.687	350	349, 332, 304	0.618

## Supplementary Materials

Supplementary materials can be accessed at: http://www.mdpi.com/1420-3049/21/6/652/s1.
