# Supplementary material for: Bioactive Constituents of Zanthoxylum rhetsa Bark and Its Cytotoxic Potential against B16-F10 Melanoma Cancer and Normal Human Dermal Fibroblast (HDF) Cell Lines
Source: Molecules. 2016 May 24;21(6):652. doi: 10.3390/molecules21060652 (PMC6274200; doi:10.3390/molecules21060652)
Supplement: Supplementary file 1 [file molecules-21-00652-s001.pdf]

Supplementary Materials: Bioactive Constituents of *Zanthoxylum rhetsa* Bark and Its Cytotoxic Potential against B16-F10 Melanoma Cancer and Normal Human Dermal Fibroblast (HDF) Cell Lines

Ramesh Kumar Santhanam, Syahida Ahmad, Faridah Abas, Intan Safinar Ismail, Yaya Rukayadi, Muhammad Tayyab Akhtar and Khozirah Shaari

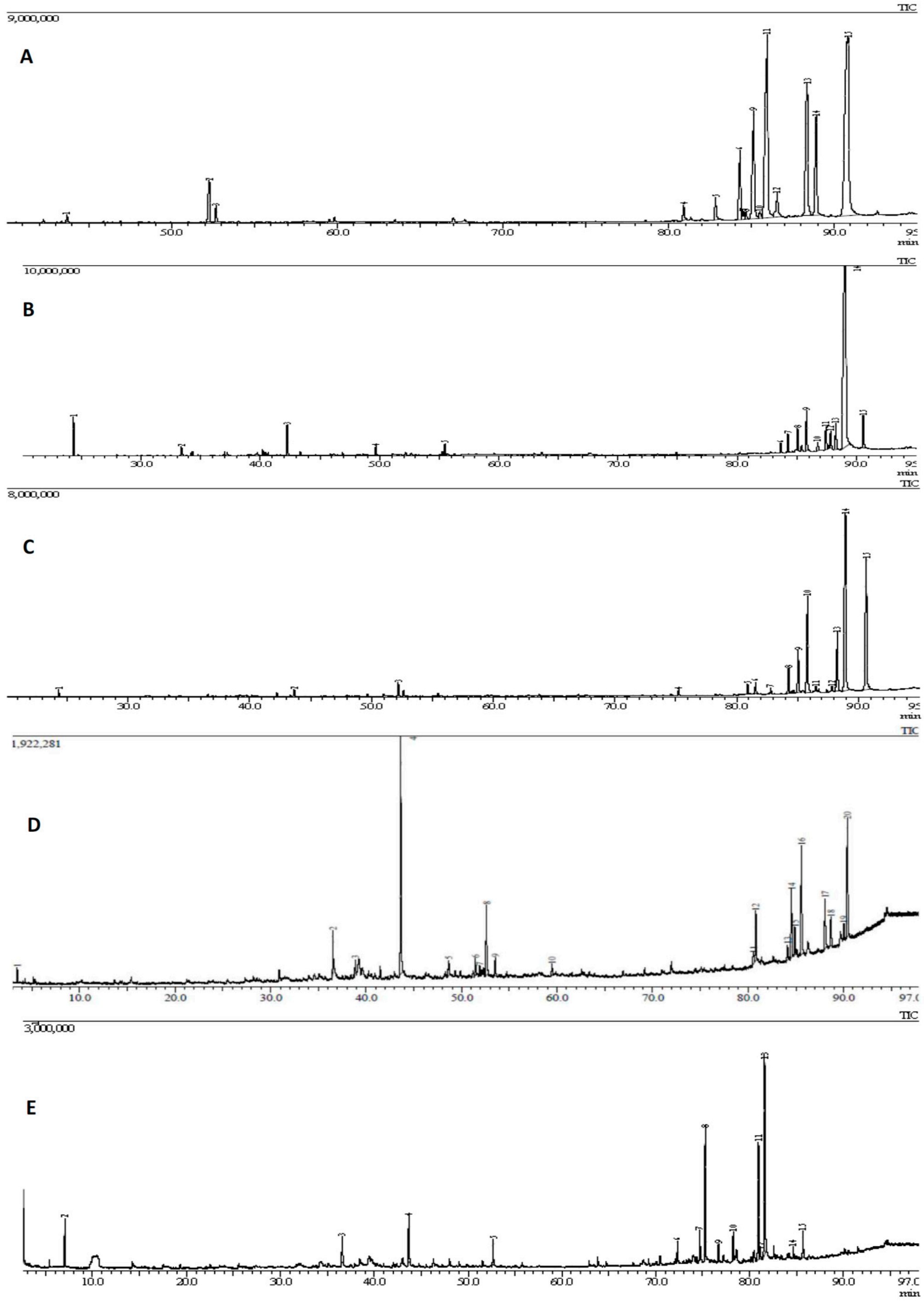

Figure S1. GC-MS Spectra of various fractions of *Z. rhetsa*: (**A**) Methanol; (**B**) Hexane; (**C**) Chloroform; (**D**) Ethyl Acetate; (**E**) Butanol.

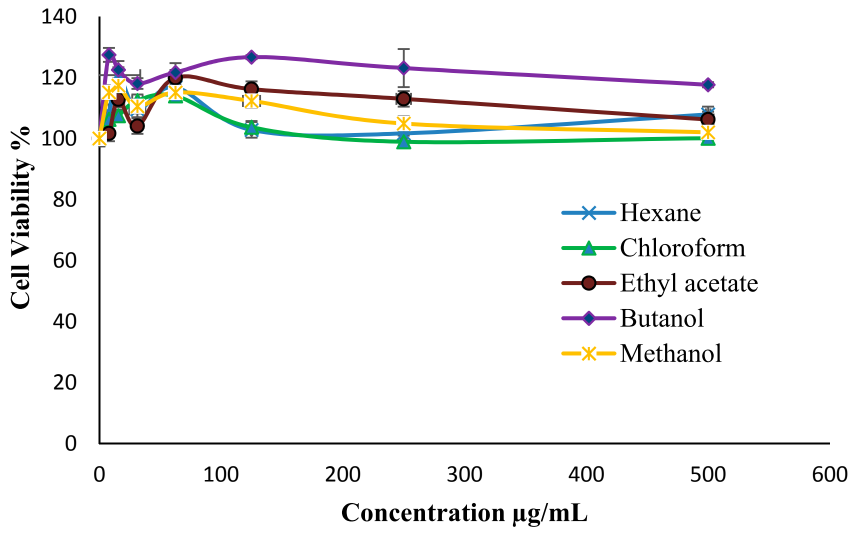

Figure S2. Cytotoxic effect of various fractions of *Z. rhetsa* at different concentration (0–500 µg/mL) against HDF cells. Data are expressed as mean ± SD of three independent experiments.

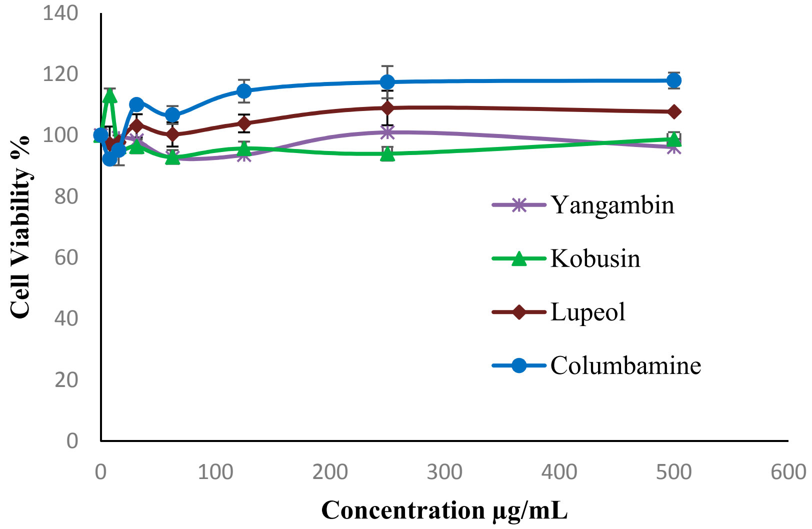

Figure S3. Cytotoxic effect of isolated compounds from *Z. rhetsa* at various concentration (0–500 µg/mL) against HDF cells. Data are expressed as mean ± SD of three independent experiments.
